# Phylogenetic analysis of the promoter element 2 of paramyxo- and filoviruses

**DOI:** 10.1128/spectrum.00417-24

**Published:** 2024-04-12

**Authors:** Shoichi Ashida, Shohei Kojima, Takashi Okura, Fumihiro Kato, Wakako Furuyama, Shuzo Urata, Yusuke Matsumoto

**Affiliations:** 1 Transboundary Animal Diseases Research Center, Joint Faculty of Veterinary Medicine, Kagoshima University, Kagoshima, Japan; 2 Genome Immunobiology RIKEN Hakubi Research Team, RIKEN Center for Integrative Medical Sciences, Yokohama, Japan; 3 Department of Virology 3, National Institute of Infectious Diseases, Tokyo, Japan; 4 National Research Center for the Control and Prevention of Infectious Diseases (CCPID), Nagasaki University, Nagasaki, Japan; Kumamoto University, Kumamoto, Japan

**Keywords:** paramyxovirus, filovirus, viral replication

## Abstract

Paramyxo- and filovirus genomes are equipped with bipartite promoters at their 3*'* ends to initiate RNA synthesis. The two elements, the primary promoter element 1 (PE1) and the secondary promoter element 2 (PE2), are separated by a spacer region that must be precisely a multiple of 6 nucleotides (nts), indicating these viruses adhere to the “rule of six.” However, our knowledge of PE2 has been limited to a narrow spectrum of virus species. In this study, a comparative analysis of 1,647 paramyxoviral genomes from a public database revealed that the paramyxovirus PE2 can be clearly categorized into two distinct subcategories: one marked by C repeats at every six bases (exclusive to the subfamily *Orthoparamyxovirinae*) and another characterized by CG repeats every 6 nts (observed in the subfamilies *Avulavirinae* and *Rubulavirinae*). This unique pattern collectively mirrors the evolutionary lineage of these subfamilies. Furthermore, we showed that PE2 of the *Rubulavirinae*, with the exception of mumps virus, serves as part of the gene-coding region. This may be due to the fact that the *Rubulavirinae* are the only paramyxoviruses that cannot propagate without RNA editing. Filoviruses have three to eight consecutive uracil repeats every six bases (UN_5_) in PE2, which is located in the 3*'* end region of the genome. We obtained PE2 sequences from 2,195 filoviruses in a public database and analyzed the sequence conservation among virus species. Our results indicate that the continuity of UN_5_ hexamers is consistently maintained with a high degree of conservation across virus species.

**IMPORTANCE:**

The genomic intricacies of paramyxo- and filoviruses are highlighted by the bipartite promoters—promoter element 1 (PE1) and promoter element 2 (PE2)—at their 3*'* termini. The spacer region between these elements follows the “rule of six,” crucial for genome replication. By a comprehensive analysis of paramyxoviral genome sequences, we identified distinct subcategories of PE2 based on C and CG repeats that were specific to *Orthoparamyxovirinae* and *Avulavirinae*/*Rubulavirinae*, respectively, mirroring their evolutionary lineages. Notably, the PE2 of *Rubulavirinae* is integrated into the gene-coding region, a unique trait potentially linked to its strict dependence on RNA editing for virus growth. This study also focused on the PE2 sequences in filovirus genomes. The strict conservation of the continuity of UN_5_ among virus species emphasizes its crucial role in viral genome replication.

## INTRODUCTION

The order *Mononegavirales* is a group of non-segmented negative-strand RNA viruses that includes the families *Rhabdoviridae*, *Paramyxoviridae*, *Pneumoviridae*, *Filoviridae*, and *Bornaviridae*, all of which contain many important animal and human pathogens that cause diseases, such as rabies, measles, respiratory syncytial virus disease, and Ebola virus disease. All viruses in the order *Mononegavirales* share a common mechanism of mRNA transcription and genome replication (1). The negative-strand RNA genome is entirely encapsidated by the viral nucleoprotein (NP), which serves as template for the viral RNA-dependent RNA polymerase (RdRp) complex composed of the large protein (L) and cofactor(s), such as phosphoprotein (P) or VP35. Although it is common that the viral genomic 3*'* terminus acts as a replication promoter (termed promoter element 1; PE1) for viral RdRp, viruses in the families *Paramyxoviridae* and *Filoviridae* possess bipartite promoters, which require a secondary promoter element (PE2) located in the internal genomic region (2–6). The paramyxo- and filoviruses employ an RNA-editing mechanism in the P gene, and in the NP, glycoprotein, and L genes, respectively (7–9). During the mRNA transcription process, viral RdRp recognizes the *cis*-acting element of the RNA-editing signal within the respective gene. Co-transcriptionally, non-template nucleotides (nts) are appended to the mRNA, enabling the synthesis of multiple proteins from a single gene (10–12). It is important to note that all viruses possessing bipartite promoters and RNA editing are inherently subject to the “rule of six,” which requires hexamer phasing of nucleotides within a genomic region to facilitate viral propagation (2–6, 13). In both paramyxo- and filoviruses, the genomic RNA is covered by NPs, with each NP monomer binding 6 nts (14–17).

**Fig 1:**
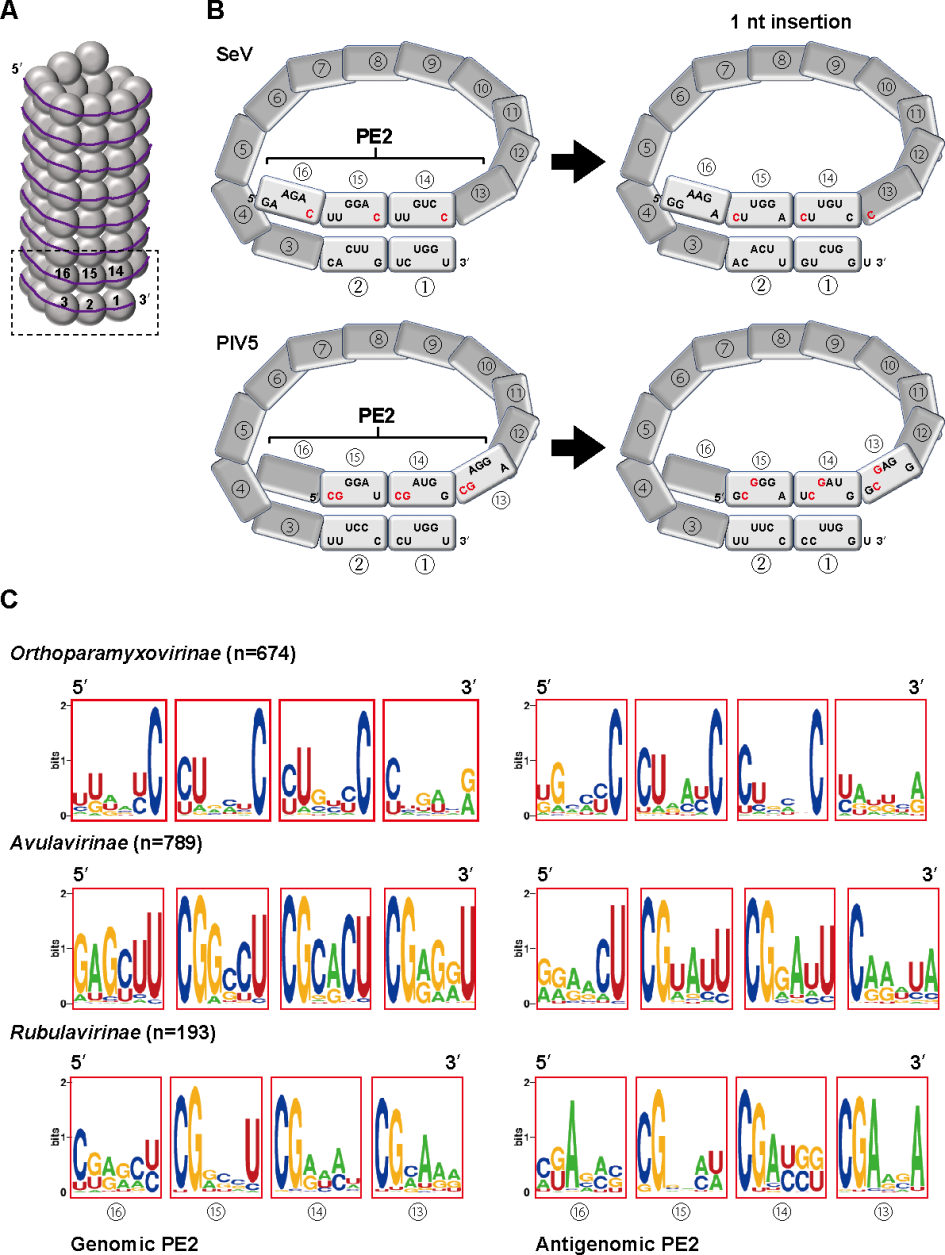
Extensive comparative analysis of the PE2 sequences of viruses in the family *Paramyxoviridae*. (**A**) Schematic of the paramyxovirus helical nucleocapsid. The dotted box indicates the replication promoters that are shown in more detail in Fig. 1B. The gray circles indicate NPs, and the numbers in the circles indicate the number of the NP hexamer from the 3*'* terminus. The line indicates RNA. (**B**) Schematic of the replication promoters for the genomic RNA of SeV and PIV5. The NPs (gray squares) bind to a sequence of 6 nts. The numbers in the circles indicate the positional number of the NP hexamer from the 3*'* terminus. The normal states are shown on the left, while the nucleotide positions when a single-nucleotide insertion has occurred upstream are shown on the right. The 24 nts covered by the 13th to the 16th NP monomer collectively constitute the PE2. The conserved nucleotides in PE2 are highlighted in red. (**C**) Conserved nucleotides within the genomic and antigenomic promoters of viruses belonging to the subfamilies *Orthoparamyxovirinae*, *Avulavirinae*, and *Rubulavirinae*. The numbers in the circles below the diagrams indicate the number of the hexamer from the 3*'* terminus.

The bipartite replication promoters play an essential role in that the virus genome is recognized as the multiple of 6 by RdRp of paramyxoviruses. The paramyxoviral PE2 is characterized by the presence of specific nucleotides at every 6 nts. Paramyxovirus nucleocapsids have 13 NP subunits per turn such that PE1 and PE2 are juxtaposed on the same face of the nucleocapsid helix for concerted recognition by the viral RdRp (7, 8). In Sendai virus (SeV) in the subfamily *Orthoparamyxovirinae*, the 14th to 16th hexamers contain 5′-GAAGAC UUGGAC UUGUCC-3′, in which the 6th nt is always a C (Fig. 1A and B, PE2) (2). In parainfluenza virus type 5 (PIV5) in the subfamily *Rubulavirinae*, the 13th to 15th hexamers contain 5′-CGGGAU CGAUGG CGAGGA-3′, in which the 5′-proximal 2 nts are always CG (Fig. 1A and B, PE2) (4). If a 1-nt insertion occurs upstream in the genome, the repeated C in SeV and the repeated CG in PIV5 would be shifted by 1 nt (Fig. 1B, 1-nt insertion). In this case, viral RdRp cannot recognize the genome as a correct template, resulting it to non-template for further replication, which acts to keep the remaining genome in a multiple of 6 in the infected cells. Although the PE2 is thus essential for viral genome replication, its characteristics have only been studied in limited numbers of virus species.

The family *Filoviridae* contains several genera, including *Ebolavirus*, *Marburgvirus,* and *Cuevavirus*. The genus *Ebolavirus* contains at least six viruses, including Zaire ebolavirus (ZEBOV), Sudan ebolavirus (SUDV), Tai forest ebolavirus (TAFV), Reston ebolavirus (RESTV), Bundibugyo ebolavirus (BDBV), and Bombali ebolavirus (BOMBV), which have been shown to have different levels of lethality in humans. The genus *Marburgvirus* includes Marburgvirus (MARV) and Ravn virus (RAVV). The characteristics of the PE2 of *Filoviridae* have been studied in detail by using ZEBOV as a representative model system. The ZEBOV genome contains PE2 in the NP mRNA 5*'* untranslated region (UTR) with uracil (U) every 6 nts (UN_5_) eight times starting from the 81st nt upstream of the genomic 3*'*-terminus (as shown in Fig. 4B) (5, 18). In contrast to the PE2 of ebolaviruses, the genus *Marburgvirus*, specifically MARV, exhibits a distinctive PE2 characteristic: UN_5_ hexamer continuity from the gene start (GS) signal (19). To date, there has been a lack of comprehensive studies on PE2 across strains of *Filoviridae*, leaving the extent of the diversity within PE2 among these viruses unknown.

In this study, we comprehensively analyzed PE2 sequences using a public database and found that almost all paramyxoviruses have C or CG repeats in PE2; the sequence patterns in PE2 were clearly divided in a subfamily-specific manner. The comparable analysis for the position of PE2 and virological properties of the genome replication indicates a unique characteristic specific for the *Rubulavirinae*. The filovirus PE2 is composed of consecutive UN_5_ hexamers, and we confirmed that the continuity of UN_5_ is unique to each virus species (three to eight times) with a high degree of conservation.

## RESULTS

### Comparison of PE2 sequences in the family *Paramyxoviridae*

For the comprehensive analysis of PE2 sequences across paramyxoviruses, complete genome sequences annotated as “Paramyxoviridae” were downloaded from the NCBI refseq database, yielding a total of 5,100 sequences. The sequence length ranged from 13,488 nt (Avian orthoavulavirus 1, MK495880.1) to 20,544 nt (Ninove microtus virus, OK623355.1). Within this data set, 1,647 sequences exhibited the conserved paramyxoviral genome terminus 5′-ACC-GGU-3′, and notably, 99.3% of these sequences conformed to a multiple of six length (Table S1). Further classification of the sequences revealed three subfamilies, *Orthoparamyxovirinae*, *Avulavirinae,* and *Rubulavirinae*, consisting of 675, 789, and 183 sequences, respectively. To assess sequence conservation, we examined the 73 to 98 nts from the 3*'* end of the genome, which represent the genomic PE2, and from the 3*'* end of the antigenome, which represent the antigenomic PE2, using the WebLogo sequence logo generator (https://weblogo.berkeley.edu/logo.cgi). Almost all viruses in the subfamily *Orthoparamyxovirinae* possess a conserved PE2 sequence pattern characterized by a C every 6 nts (N_5_C) in the hexamers number (hex#) 14, 15, and 16 within both the genomic and antigenomic PE2 (Fig. 1C). In contrast, in the majority of the *Avulavirinae* and *Rubulavirinae* viruses, CG was found in every 6 nts (CGN_4_) in hex# 13, 14, and 15 in both the genomic and antigenomic PE2 (Fig. 1C). While some variation emerged within virus genera (Fig. S1), it was clear that PE2 sequence conservation could be definitely categorized into two patterns: N_5_C for hex# 14, 15, and 16 (*Orthoparamyxovirinae*) and CGN_4_ for hex# 13, 14, and 15 (*Avulavirinae* and *Rubulavirinae*).

### Differences in the antigenomic PE2 location among paramyxovirus subfamilies

**Fig 2:**
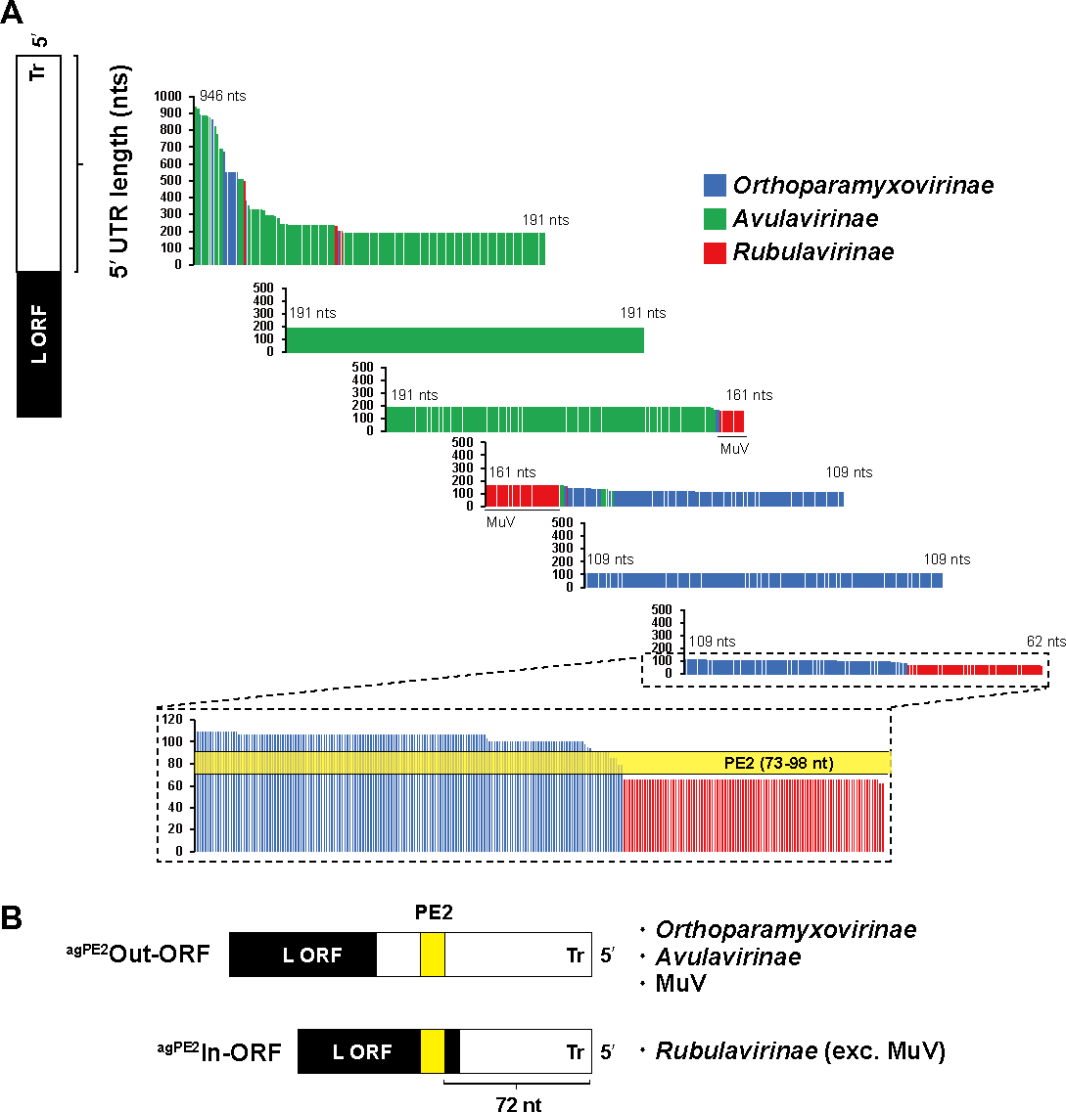
Analysis of the genomic 5*'* UTR lengths of viruses in the family *Paramyxoviridae*. (**A**) The nucleotide length of the genomic 5*'* UTRs of viruses belonging to the subfamilies *Orthoparamyxovirinae*, *Avulavirinae*, and *Rubulavirinae*. Each thin vertical line represents a virus sequence. The antigenomic PE2 (agPE2) region (nts 73 to 98) is shown in yellow. (**B**) Viruses are divided into agPE2 Out- or In-ORF types. Tr indicates trailer sequence.

The genomic PE2 is presumed to reside within the 3*'* UTR of the genome, while the antigenomic PE2 is situated in the 5*'* UTR of the genome. However, our previous investigations have revealed that in the human parainfluenza virus type 2 belonging to the subfamily *Rubulavirinae*, the antigenomic PE2 is localized within the open reading frame (ORF) of the viral L protein (20). Consequently, paramyxoviruses may be categorized into two distinct classes: those harboring PE2 outside of the ORF and those harboring PE2 within the ORF. By examining the length of the 3*'* UTR and 5*'* UTR of each viral genome, it is possible to determine whether PE2, which is located at position 73 to 98 from the genomic and antigenomic ends, is situated outside or inside of the ORF. We first comprehensively examined the lengths of the 3*'* UTRs in 1,647 genome sequences of paramyxoviruses (Fig. S2). The shortest 3*'* UTR was 98 nts in the Denwin virus in the subfamily *Orthoparamyxovirinae* (OK623354), indicating that there are no viruses in the database with a genomic PE2 within the ORF. Subsequently, the lengths of the 5*'* UTRs of the genomes were comprehensively examined in the same database. The findings indicated that all viruses belonging to the subfamily *Rubulavirinae*, with some exceptions, e.g., mumps virus (MuV), had antigenomic PE2 sequences embedded within the L ORF (Fig. 2A and B). Furthermore, we observed that the antigenomic PE2 sequences in only 15 viruses of the subfamily *Orthoparamyxovirinae* were partially within the L ORF (Table S1). For all of the other viruses, the antigenomic PE2 sequences were located outside of the ORF.

**Fig 3:**
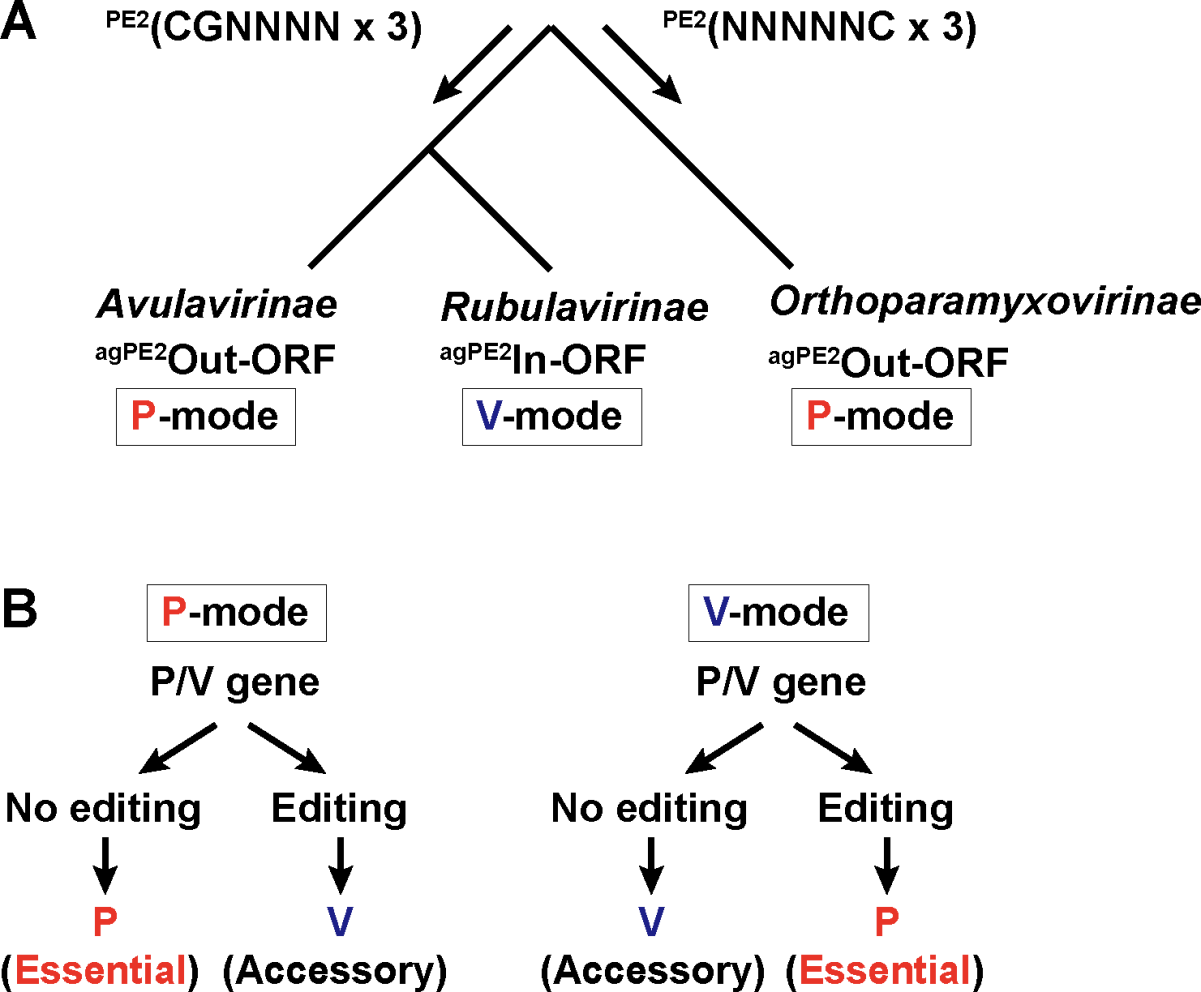
Relationship between PE2 and the virological properties of paramyxoviruses. (**A**) Relationships within the evolutionary phylogenetic tree, the sequence and location of PE2, and the RNA-editing pattern. (**B**) Differences in the RNA-editing modes. The P mode produces an accessory protein via editing, whereas the V mode produces an essential P protein via editing.

The viruses within the family *Paramyxoviridae* are categorically delineated, as illustrated in Fig. 3A, based on a phylogenetic tree deduced from the alignment of L proteins (21). The evolutionary trajectory shows an initial divergence of the viruses into an *Orthoparamyxovirinae* lineage and another lineage that subsequently splits into the *Avulavirinae* and *Rubulavirinae* lineages. The initial divergence is strikingly consistent with the divergence of the N_5_C PE2 and CGN_4_ PE2 patterns (Fig. 3A). The *Rubulavirinae* lineage is characterized by the localization of the antigenomic PE2 within the ORF and by a different RNA-editing mechanism (V-mode) (Fig. 3A). P-mode viruses perform RNA editing to generate the accessory V gene, while V-mode viruses perform RNA editing to generate the essential P gene (Fig. 3B) (22–27).

### Comparison of genomic PE2 sequences in the family *Filoviridae*

**Fig 4:**
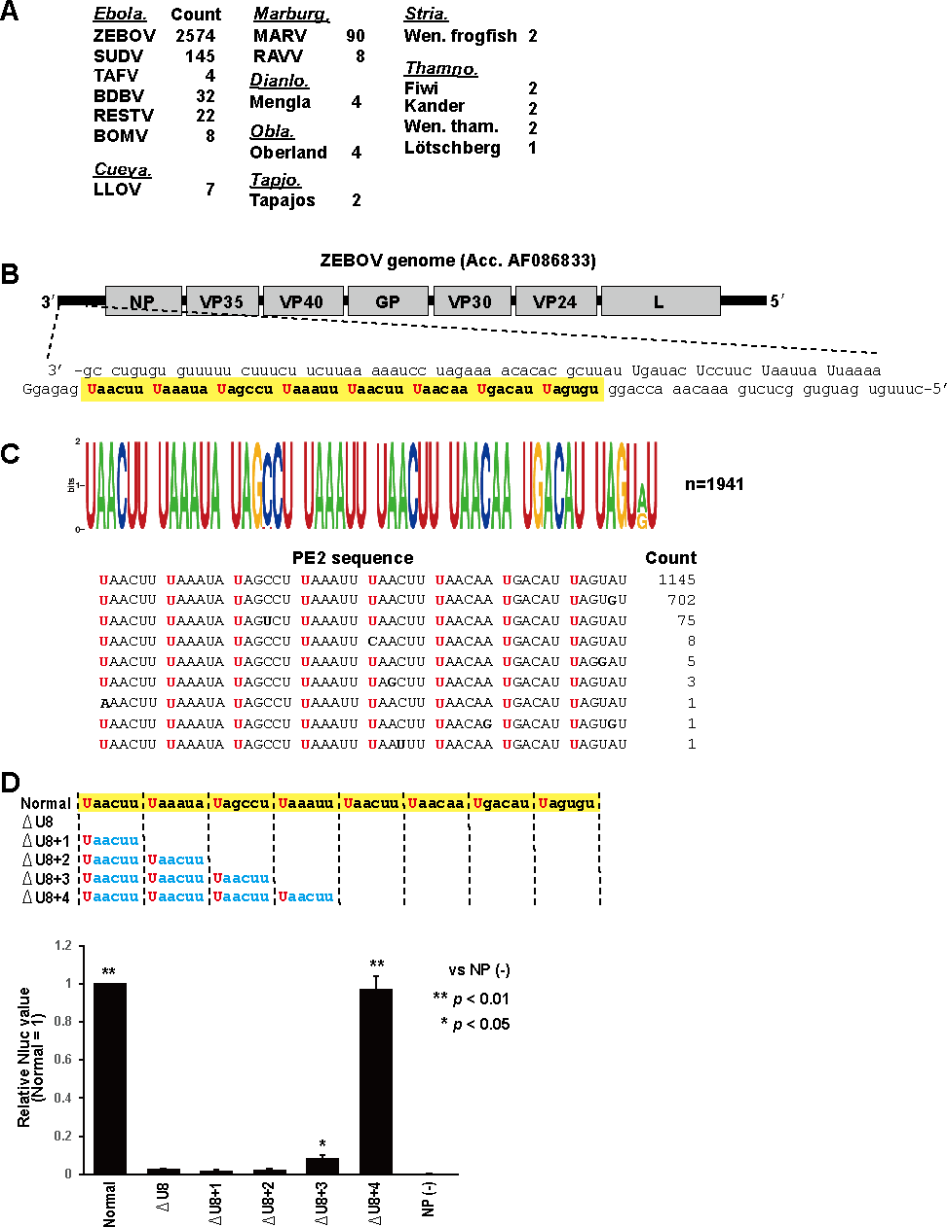
Comprehensive analysis of filovirus PE2 sequences. (**A**) Sequence counts used in the analysis of PE2 sequences. The virus genera are shown in italic letters. (**B**) The PE2 sequence of ZEBOV. The 3′ UTR sequence from a representative ZEBOV genome (AF086833) is shown. (**C**) The PE2 sequences of ZEBOV. All sequences found in the data set are shown below. (**D**) The minigenome study of ZEBOV. The relative values of Nluc expression are shown with the values of the normal ZEBOV minigenome set to 1. NP (−) indicates the value from the assay using an empty plasmid instead of the plasmid encoding ZEBOV NP. Bars represent the means and standard deviations (*n* = 3 from three independent experiments).

Ebolavirus in the family *Filoviridae* follows the rule of six, which is limited to the genomic promoter portion, and has PE2, similar to paramyxoviruses (5, 6). To examine the genomic PE2 sequences of filoviruses in detail, the “Filoviridae” genome sequences were downloaded from the NCBI refseq database, yielding 4,664 sequences. After excluding artificially generated and unverified sequences, a total of 2,905 sequences were obtained. The length of the sequences ranged from 13,066 nt (Kander virus, NC_076916.1) to 19,151 nt (MARV, AY358025.2). The 2,905 sequences included sequences from the following genera: *Ebolavirus*, ZEBOV (2,574 sequences), SUDV (145 sequences), TAFV (4 sequences), BDBV (32 sequences), RESTV (22 sequences), and BOMV (6 sequences); *Cuevavirus*, LLOV (7 sequences); *Marburgvirus*, MARV (90 sequences) and RAVV (8 sequences); and others, e.g., *Dianlovirus*, *Oblavirus*, *Tapjovirus*, *Striavirus,* and *Tamnovirus* as shown in Fig. 4A.

The genomic PE2 of ebolaviruses has been studied in detail, especially in ZEBOV (5). The 3*'* UTR of a ZEBOV genome sequence (AF086833) is shown in Fig. 4B as a representative sequence. The ZEBOV PE2 is located in the NP mRNA region with U at every 6 nts eight times. We searched for a sequence similar to the PE2 of ZEBOV (AF086833) at the 3*'* end of the genome in all 2,574 sequences of ZEBOV obtained. Among the 2,574 sequences, PE2 could not be confirmed in 633 sequences because the 3*'* genomic end containing the PE2 region was not registered in the database. For the remaining 1,941 sequences, the PE2 sequence was identified, and sequence homology was examined using the WebLogo sequence logo generator (Fig. 4C). PE2 homogeneity was confirmed in these sequences, with 1,145 sequences perfectly matching that of AF086833. The other 796 sequences displayed minor mutations in the PE2 region (Fig. 4C). Notably, only 9 of 1,941 sequences exhibited mutations in the U position in the UN_5_ hexamers. One sequence had the first U from the 3′ end changed to A (MG572232.1, isolated in Guinea in 2014), while eight other sequences had the fifth U from the 3′ end changed to C (LT630506.1, LT630508.1, LT630510.1, LT630513.1, LT630521.1, LT630541.1, LT630547.1, and LT630586.1, isolated in Guinea in 2015). These eight PE2 sequences are separated into four and three consecutive UN_5_ hexamers.

Weik et al. had shown in a minigenome system that three consecutive UN_5_ hexamers located in the proper phase in PE2 were sufficient for ZEBOV minigenome activity (5). They suggested that replication occurs more efficiently when more hexamers are present. We also used the ZEBOV minigenome system to confirm that the deletion of eight consecutive UN_5_ hexamers eliminated the minigenome activity (Fig. 4D). Using minigenomes with 6 nts containing a leading U (Uaacuu) added to the deletion region one hexamer at a time, it was shown that three consecutive occurrences of Uaacuu resulted in slight recovery of the minigenome activity, while four consecutive occurrences of Uaacuu resulted in complete recovery of the activity (Fig. 4D). Consequently, a PE2 sequence divided into four and three consecutive UN_5_ hexamers may exhibit no significant impact on viral polymerase activity, as evidenced by the minigenome system.

**Fig 5:**
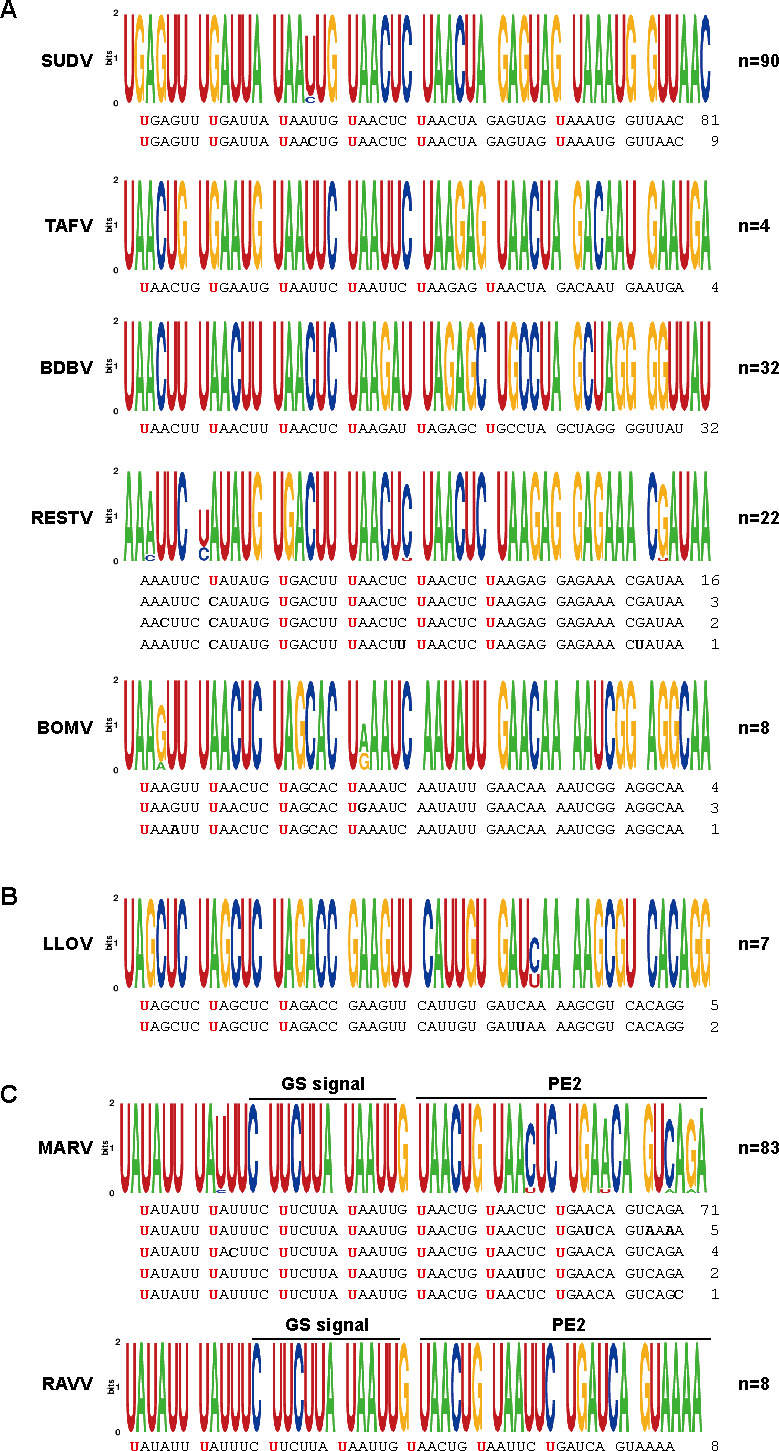
Comprehensive analysis of filovirus PE2 sequences other than ZEBOV. (**A**) The PE2 sequences of SUDV, TAFV, BDBV, RESTV, and BOMBV (genus *Ebolavirus*). (**B**) The PE2 sequences of LLOV (genus *Cuevavirus*). (**C**) The PE2 sequences of MARV and RAVV (genus *Marburgvirus*).

We subsequently performed a comparative analysis of genomic PE2 sequences in ebolavirus species other than ZEBOV (Fig. 5A). Among 90 SUDV sequences, only two PE2 patterns were identified, each characterized by the consistent presence of five consecutive UN_5_ hexamers. TAFV, with only four genome sequences in the database, exhibited uniform PE2 sequences featuring six consecutive UN_5_ hexamers. The 32 BDBV genome sequences in the database all exhibited uniform PE2 sequences featuring six consecutive UN_5_ hexamers. Within the 22 RESTV sequences, two PE2 patterns were found—one comprised 16 sequences of five consecutive UN_5_ hexamers, and the other comprised six sequences of four consecutive UN_5_ hexamers. In the eight BOMBV genome sequences in the database, all PE2 sequences exhibited four consecutive UN_5_ hexamers. These findings showed the conservation of four or more consecutive UN_5_ hexamers in the PE2 sequences across all ebolavirus species.

Seven LLOV sequences within the genus *Cuevavirus* were found in the database, and they contained three consecutive UN_5_ hexamers within PE2 (Fig. 5B). No sequences with more than four consecutive UN_5_ hexamers were identified in the 3′ UTR of the LLOV genomes. MARV and RAVV of the genus *Marburgvirus* exhibit a distinctive PE2 characteristic: UN_5_ hexamers continuity from the GS signal (19). Analysis of the homology between the GS-containing portion and PE2 in MARV and RAVV revealed that all 83 MARV PE2 sequences had 7 consecutive UN_5_ hexamers, with no observed mutations in the U (Fig. 5C). All eight RAVV PE2 sequences were identical to one of the MARV PE2 sequences.

## DISCUSSION

We compared the PE2 sequences, which have only been studied in detail in a limited number of virus species, using a database containing all available paramyxovirus genome sequences. The results showed that the PE2 of paramyxoviruses can clearly be classified into two groups: those with N_5_C at hex# 14, 15, and 16 (*Orthoparamyxovirinae*) and those with CGN_4_ at hex# 13, 14, and 15 (*Avulavirinae* and *Rubulavirinae*) (Fig. 1C). This is consistent with previous findings showing the PE2 sequences of several representative paramyxoviruses (7, 8, 28). This difference in the PE2 pattern is consistent with the evolutionary phylogenetic tree of the viruses (21); the ancestors first diverged into an *Orthoparamyxovirinae* lineage and another lineage, which was linked to divergence of PE2 regions into those with three consecutive N_5_C versus three CGN_4_ hexamers (Fig. 3A). Using the lengths of the 3*'* UTR and 5*'* UTR as a simple indicator of whether PE2 is located inside or outside of the ORF, we found that most viruses in the subfamily *Rubulavirinae*, with the exception of a few viruses including MuV, have the antigenomic PE2 within the ORF of the L protein (Fig. 2*). The Rubulavirinae* lineage differs from the *Orthoparamyxovirinae* and *Avulavirinae* lineages in that the P mRNA is produced by RNA editing (23, 26, 27). While the *Rubulavirinae* are strictly reliant on RNA editing for viral propagation, the *Orthoparamyxovirinae* and *Avulavirinae* require it only for accessory protein production (22, 24, 25). RNA-editing efficiency is regulated by an RNA-editing signal present on the P gene that functions as a *cis*-acting element (7), and NPs also bind to this *cis*-acting element region. Notably, the positioning of the *cis*-acting element on NPs affects the RNA-editing efficiency (29). When NPs bind correctly from the 5*'* end of the genome, the *cis*-acting element is placed correctly on the NPs (7, 29). The antigenomic PE2 functions as a promoter during genome replication from the antigenome, and the precise placement of the *cis*-acting element on NP relies on whether the spacer sequence between the antigenomic PE1 and antigenomic PE2 is a multiple of 6 (7, 29). Therefore, if there is no mutation in PE2, the insertion/deletion-sensing system of the genome can operate correctly, and the *cis*-acting element will be placed correctly on the NP. The non-coding regions are more susceptible to mutations than the coding regions. Particularly, the ORF for the essential RdRp L protein has remained remarkably conserved throughout viral evolution. The *Rubulavirinae*, which have an antigenomic PE2 embedded within the L ORF, appear to have evolved mechanisms to resist insertions, deletions, and other mutations in PE2 more effectively than viruses of the other subfamilies. Further research is warranted to determine conclusively whether there is any connection between the RNA-editing efficiency and the antigenomic PE2 sequence.

In this study, we systematically searched a public database for all filovirus sequences and investigated the PE2 sequences in viruses within the genera *Ebolavirus*, *Cuevavirus,* and *Marburgvirus*. A total of 1,941 PE2 sequences were acquired for ZEBOV, including those isolated from 1976 to 2020 in various regions, such as the Democratic Republic of the Congo, Gabon, Guinea, Liberia, Mali, Nigeria, Republic of Congo, Sierra Leone, and Uganda (Fig. 4C; Table S2). Despite the diverse geographic origins of the isolates, the ZEBOV PE2 sequences showed remarkable uniformity. The PE2 sequences of viruses of the genus *Ebolavirus* other than ZEBOV also showed no significant differences in the PE2 sequences among virus strains. The PE2 of viruses in the genus *Marburgvirus* exhibits a distinct feature with seven consecutive UN_5_ hexamers, including the GS signal (19, 30); this UN_5_ continuity was fully conserved in all 91 sequences including both MARV and RAVV (Fig. 5C). The continuity of UN_5_ hexamers is fixed for each virus species in filoviruses with an extremely high degree of conservation.

The PE2 sequences within the genus *Ebolavirus* exhibit variations in the continuity of UN_5_ hexamers: ZEBOV displays eight, TAFV and BDBV display six, SUDV displays five, RESTV displays five or four, and BOMBV displays four consecutive UN_5_ hexamers in their PE2. Notably, all ebolavirus species, despite their diversity, harbor four or more consecutive UN_5_ hexamers in PE2. Our ZEBOV minigenome experiment showed that a minimum of four consecutive UN_5_ hexamers is imperative for optimal minigenome activity (Fig. 4C). The necessity of four or more consecutive UN_5_ hexamers for replication appears to be a shared attribute among viruses within the genus *Ebolavirus*. The majority of the PE2 sequences in ZEBOV are characterized by eight consecutive UN_5_ hexamers, although rare instances of altered UN_5_ hexamer continuity appear to occur in nature (Fig. 4C). We identified one sequence with a mutation in the U of the first 3′ UN_5_ hexamer that resulted in seven consecutive UN_5_ hexamers. Additionally, eight sequences displayed a mutation in the U at the midpoint of the UN_5_ hexamers, leading to a division of PE2 into four and three consecutive UN_5_ hexamers. Viruses with a longer UN_5_ hexamer repeat in PE2, such as ZEBOV, may be more resistant to mutations in the essential U since viral genome replication appears to be preserved if at least four consecutive UN_5_ hexamers are maintained.

Viruses employing an RNA-editing mechanism during mRNA transcription concurrently have bipartite promoters. Paramyxovirus RdRp recognizes the consecutive C sequence within the RNA-editing site on the P gene as a *cis*-acting element and inserts G(s) into the mRNA during the transcription process (7, 8). Filovirus RdRp recognizes the contiguous U sequence within the RNA-editing site on the NP, glycoprotein, and L genes and inserts A(s) into the mRNA (9). The PE2 of paramyxoviruses encodes C and that of filoviruses U residues every 6 nt. Thus, there appears to be a mechanism whereby paramyxovirus RdRp recognizes C-based elements, and filovirus RdRp recognizes U-based elements.

## MATERIALS AND METHODS

### Cells

Baby hamster kidney (BHK) cells constitutively expressing T7 RNA polymerase (BHK/T7-9 cells) (31) were cultured in Dulbecco Modified Eagle’s Medium with 5% fetal calf serum and penicillin/streptomycin. Cells were cultured at 37°C in 5% CO_2_.

### Plasmid construction

The nanoluciferase (Nluc)-expressing ZEBOV minigenome plasmid (ZEBOV-Nluc) was constructed using the pUC57 plasmid backbone. The Nluc gene was amplified by PCR and flanked by the 3*'* UTR (469 nts) containing the leader sequence and the 5*'* UTR (740 nts) containing the trailer sequence of the ZEBOV genome. The minigenome is set under the control of the T7 RNA polymerase promoter, and the transcript expressed as a negative-sense RNA is cleaved at both ends by a hammerhead ribozyme and a hepatitis delta virus ribozyme (32). The ZEBOV NP, VP35, VP30, and L genes cloned into a pCAGGS vector were as described previously (33). A pCAGGS-derivative plasmid for the expression of firefly luciferase (Fluc) was also constructed. The deletions of the PE2 in ZEBOV-Nluc were performed by a standard cloning method. Briefly, ZEBOV-Nluc lacking 8, 7, 6, 5, and 4 sets of the eight consecutive UN_5_ hexamers (ΔU8, ΔU8 + 1, ΔU8 + 2, ΔU8 + 3, and ΔU8 + 4, respectively) within PE2 were amplified by PCR and cloned into the pUC57 plasmid. The virus sequence used in this study was derived from a ZEBOV strain (GenBank accession number AF086833).

### ZEBOV-Nluc minigenome assay

The ZEBOV-Nluc minigenome assay was performed in BHK/T7-9 cells cultured in 12-well plates (1 × 10^5^ cells/well). The plasmids ZEBOV-Nluc (0.5 µg), pCAGGS-L (0.4 µg), -VP35 (0.4 µg), -VP30 (0.4 µg), and -NP (0.4 µg) or empty vector, and Fluc (0.05 µg) were transfected using XtremeGENE HP (Merck, Darmstadt, Germany). At 48 h post-transfection, the cells were lysed with passive lysis buffer (Promega, Madison, WI, USA), and the Nluc and Fluc activities were measured using the Nano-Glo Dual-Luciferase Reporter Assay System (Promega) according to the manufacturer’s instructions. All activity values measured for Nluc were normalized to the expression levels of Fluc.

### Analysis of the PE2 sequences and UTR lengths of the viruses in the family *Paramyxoviridae*

Sequences annotated as Paramyxoviridae were downloaded from the NCBI refseq database on 3 August 2022. There were 64,057 sequences annotated as Paramyxoviridae in the database. Based on the information that the length of paramyxovirus sequences ranged from 13,488 nt (Avian orthoavulavirus 1, MK495880.1) to 20,544 nt (Ninove microtus virus, OK623355.1), sequences shorter than 10,000 nt and longer than 30,000 nt (*n* = 58,957) were excluded from the analysis. Within the obtained data set, 1,647 sequences exhibited the conserved paramyxoviral genome terminus 5′-ACC-GGU-3′, and 99.3% of these sequences conformed to a multiple of six length. For each sequence, we detected the genome length, 3′ UTR and 5*'* UTR lengths, and extracted the sequences of the antigenomic PE2 (73 to 96 nts from the 3*'* terminus of the antigenome) and genomic PE2 (73 to 96 nts from the 3*'* terminus of the genome) (Table S1). The codes used for this analysis are available on GitHub (https://github.com/shohei-kojima/Filo_Paramyxo_2023).

### Search for PE2 sequences in the genome of viruses in the family *Filoviridae*

Sequences annotated as Filoviridae were downloaded from the NCBI refseq database on 15 February 2024. The codes used for this analysis are available on GitHub (https://github.com/shohei-kojima/Filo_Paramyxo_2023). There were 4,664 sequences annotated as Filoviridae in the database. The presence of the PE2 sequence was searched for through alignment with the PE2 sequence of each virus as previously reported (6, 30).

### Statistical analysis

Statistical analyses were performed with the Prism software (version 9.1.2; GraphPad, San Diego, CA, USA). Statistical significance was assigned when *P* values were <0.05. Inferential statistical analysis was performed by one-way analysis of variance followed by Tukey’s test.

## Supplementary Material

Reviewer comments

## Data Availability

All databases used in this study are available from DDBJ/ENA/GenBank (https://www.ddbj.nig.ac.jp/about/insdc-e.html). The accession numbers of the virus sequences used in this study are listed in Table S1 and S2.

## SUPPLEMENTAL MATERIAL

Supplemental material spectrum.00417-24-s0001.pdf Fig. S1 and S2.

Table S1 spectrum.00417-24-s0002.xlsx Paramyxovirus sequences.

Table S2 spectrum.00417-24-s0003.xlsx Filovirus sequences.

OPEN PEER REVIEW reviewer-comments.pdf An accounting of the reviewer comments and feedback.

ASM does not own the copyrights to Supplemental Material that may be linked to, or accessed through, an article. The authors have granted ASM a non-exclusive, world-wide license to publish the Supplemental Material files. Please contact the corresponding author directly for reuse.
