# Supplementary material for: Phylogenetic analysis of the promoter element 2 of paramyxo- and filoviruses
Source: Microbiol Spectr. 2024 Apr 12;12(5):e00417-24. doi: 10.1128/spectrum.00417-24 (PMC11064532; doi:10.1128/spectrum.00417-24)
Supplement: Supplemental material — Fig. S1 and S2. [file spectrum.00417-24-s0001.pdf]

Supplemental Materials

Figure legends

Supplementary Figure 1. Comparative analysis of PE2 sequence conservation within each genus of the family *Paramyxoviridae*.

Conserved nts within the genomic and antigenomic promoters of viruses belonging to the genus *Morbillivirus, Respirovirus* and *Henipavirus* (subfamily *Orthoparamyxovirinae*), *Orthoavulavirus* and *Metaavulavirus* (subfamily *Avulavirinae*), and *Orthorubulavirus* and *Pararubulavirus* (subfamily *Rubulavirinae*). The numbers in the circles below the diagrams indicate the number of the hexamer from the 3' terminus. Each red frame indicates a hexamer bound by an NP monomer.

Supplementary Figure 2. Analysis of the genomic 3' UTR lengths of viruses in the family *Paramyxoviridae*.

Nucleotide lengths of the genomic 3' UTRs of viruses belonging to the subfamilies *Orthoparamyxovirinae*, *Avulavirinae* and *Rubulavirinae*. Each thin vertical line represents a virus sequence. The genomic PE2 (gPE2) region (nts 73 to 98) is shown in yellow. Le indicates leader sequence.

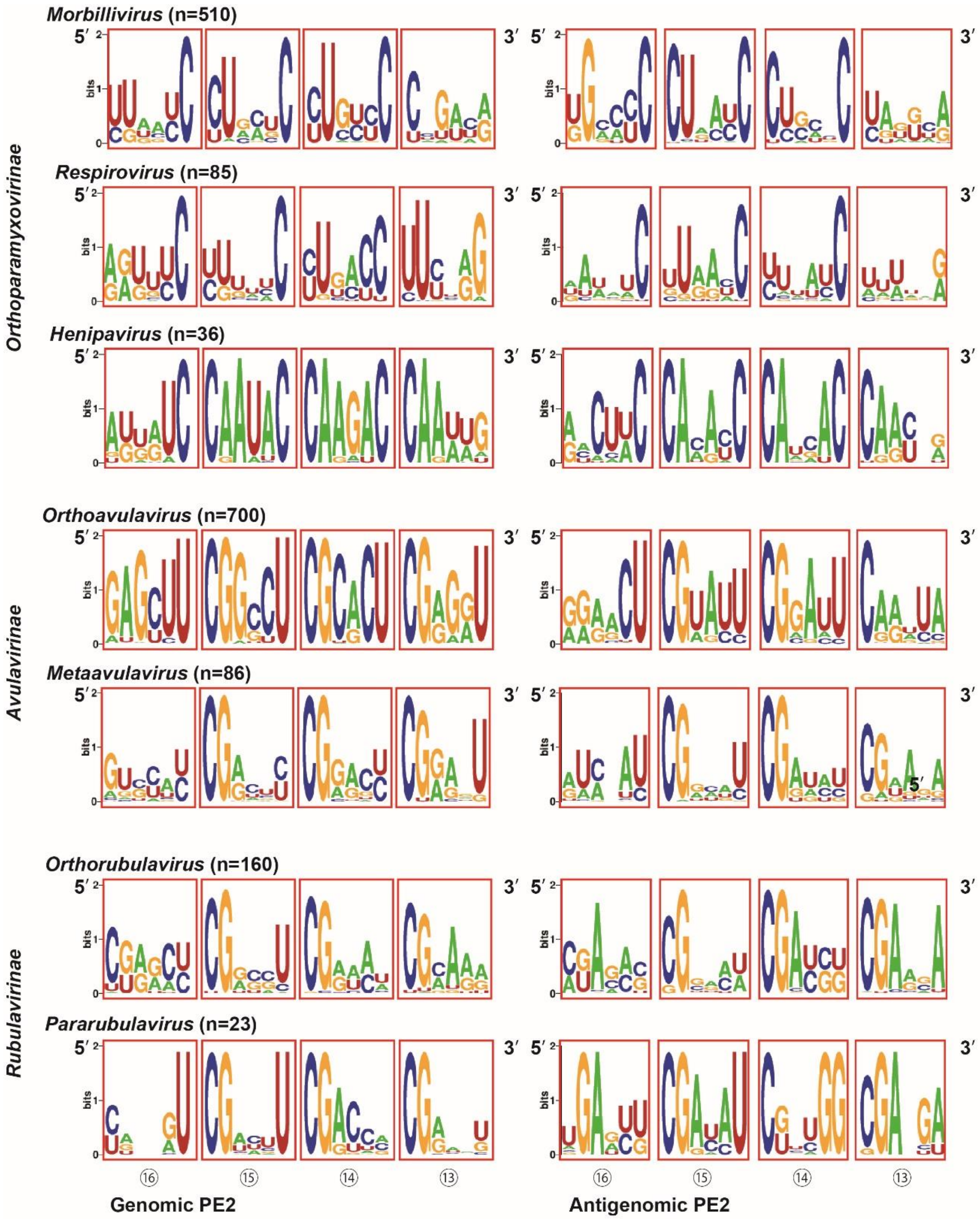

Supplementary Figure 1

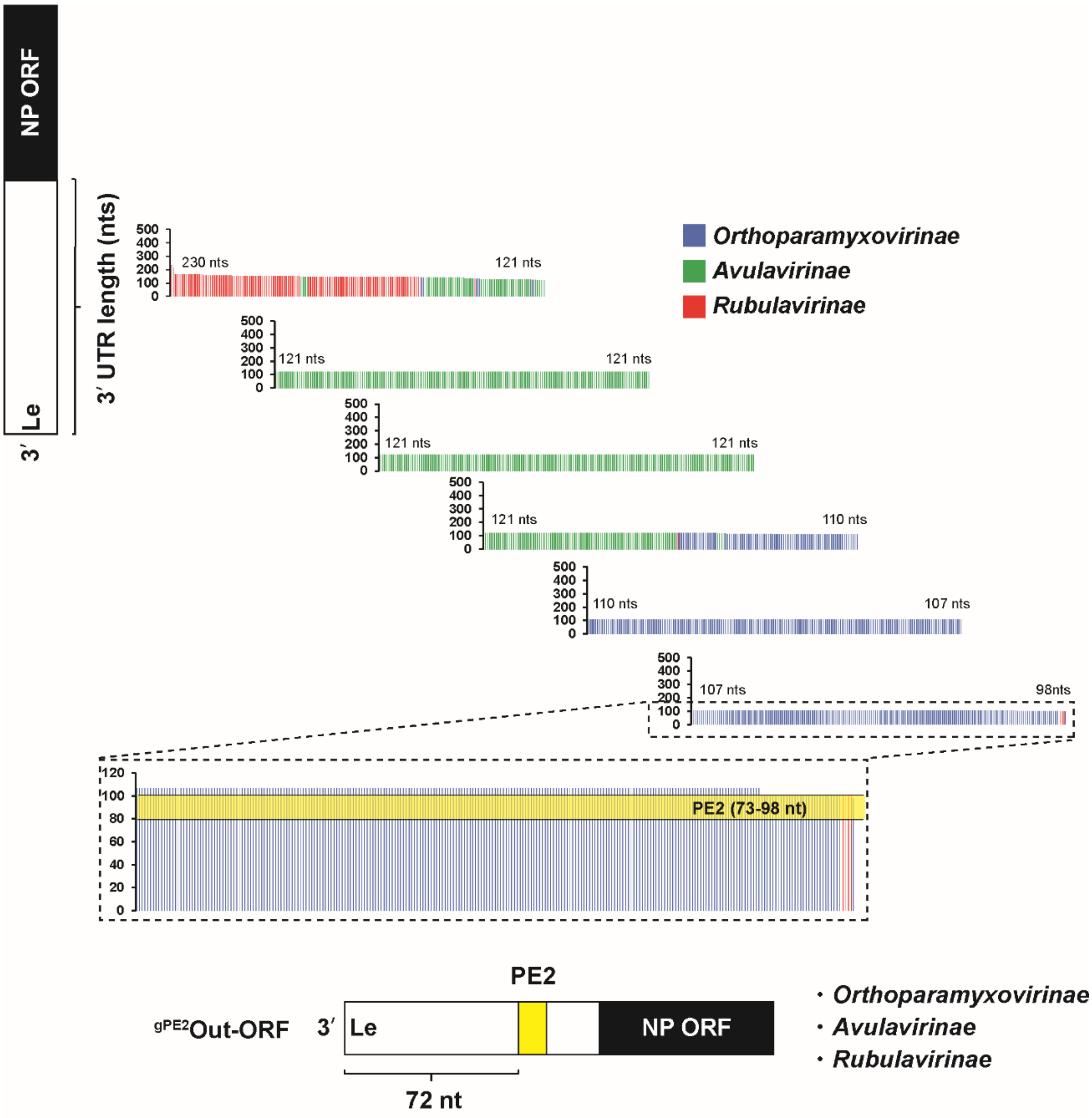

Supplementary Figure 2
